# Use of hydraulic traits for modeling genotype‐specific acclimation in cotton under drought

**DOI:** 10.1111/nph.16751

**Published:** 2020-07-18

**Authors:** Diane R. Wang, Martin D. Venturas, D. Scott Mackay, Douglas J. Hunsaker, Kelly R. Thorp, Michael A. Gore, Duke Pauli

**Affiliations:** ^1^ Department of Geography University at Buffalo Buffalo NY 14261 USA; ^2^ School of Biological Sciences University of Utah Salt Lake City UT 84112 USA; ^3^ US Arid‐Land Agricultural Research Center Maricopa AZ 37860 USA; ^4^ Plant Breeding and Genetics Section School of Integrative Plant Science Cornell University Ithaca NY 14853 USA; ^5^ School of Plant Sciences University of Arizona Tucson AZ 85721 USA; ^6^ Present address: Department of Agronomy Purdue University West Lafayette IN 47907 USA

**Keywords:** ecophysiology, genetic variation, *Gossypium hirsutum*, process‐based model, soil variability

## Abstract

- Understanding the genetic and physiological basis of abiotic stress tolerance under field conditions is key to varietal crop improvement in the face of climate variability. Here, we investigate dynamic physiological responses to water stress *in silico* and their relationships to genotypic variation in hydraulic traits of cotton (*Gossypium hirsutum*), an economically important species for renewable textile fiber production.
- In conjunction with an ecophysiological process‐based model, heterogeneous data (plant hydraulic traits, spatially‐distributed soil texture, soil water content and canopy temperature) were used to examine hydraulic characteristics of cotton, evaluate their consequences on whole plant performance under drought, and explore potential genotype × environment effects.
- Cotton was found to have R‐shaped hydraulic vulnerability curves (VCs), which were consistent under drought stress initiated at flowering. Stem VCs, expressed as percent loss of conductivity, differed across genotypes, whereas root VCs did not. Simulation results demonstrated how plant physiological stress can depend on the interaction between soil properties and irrigation management, which in turn affect genotypic rankings of transpiration in a time‐dependent manner.
- Our study shows how a process‐based modeling framework can be used to link genotypic variation in hydraulic traits to differential acclimating behaviors under drought.

## Introduction

Rising incidence of extreme drought and heat events in combination with diminishing freshwater resources associated with climate change threatens the global security of plant‐based food, fiber and feed production (Coumou & Rahmstorf, 2012; Elliott *et al*., 2014; Foster *et al*., 2015; Lesk *et al*., 2016). Despite strong interest in developing crop cultivars that are resilient to water deficit, there remain many challenges to breeding and deployment of such varieties (Atlin *et al*., 2017). One challenge is that no two drought scenarios are exactly alike. Across years and sites, managed drought trials vary in the frequency and amount of precipitation, soil texture and its related water transport characteristics, and other environmental factors such as solar radiation, temperature, and wind speed. These parameters affect processes that drive the dynamic perception and response of plants to water deficit (Campbell & Norman, 2012), and varieties considered tolerant in one set of conditions may underachieve in others. Thus, the meaning of ‘drought tolerance’ is incomplete without adequate understanding of its environmental context: soil, weather, and management (Tardieu, 2012).

Efforts to unravel the genetic basis of drought response mechanisms face similar contextual difficulties. In contrast to classical physiology studies that carry out detailed phenotyping across a small number of genotypes, genetic mapping experiments must handle germplasm panels and populations that may comprise hundreds to thousands of different entries (Yu *et al*., 2008; Bandillo *et al*., 2013; Maurer *et al*., 2015). Logistically, this translates to large‐scale evaluations with field sizing requirements that can lead to high spatial variation, for example in soil texture, that interact with temporally varying climatic and management parameters. These relationships may give rise to divergent micro‐site scenarios that can ultimately impede mapping efforts through decreased signal‐to‐noise ratios for detecting genetic associations. While mitigating the effects of environmental variation in genetic studies is often addressed with statistical treatment (Asaro *et al*., 2016), empirical approaches cannot explicitly account for the non‐linear processes that mechanistically underlie a genotype's response to environmental signals.

Process‐based ecophysiological modeling is one tool that can link environmental variation (both spatial and temporal) to variability in plant performance under water deficit (Tardieu, 2012) by coupling mathematically‐formalized processes that underlie whole‐plant physiology. Models based on first principles of physics, chemistry, and biology can, in theory, extend predictions to novel environments and add mechanistic value to observations (Bouman *et al*., 1996). Of most relevance to geneticists, these models may ultimately serve to connect genotype to phenotype based on functional relationships underlying crop genotype‐by‐environment interaction (G × E). While a diversity of process‐based models have been developed specifically for crops (e.g. refer to the collection examined by Parent & Tardieu, 2014), none to our knowledge includes the explicit modeling of rhizosphere‐xylem hydraulics to address water transport, despite the key role that water movement through the soil–plant–atmosphere continuum plays in stress response to drought. Accordingly, utilization of models that include plant hydraulics in studies of agricultural species should open the door to opportunities for a deeper genetic and physiological understanding of crop stress response to drought (Venturas *et al*., 2017).

In this study, we employ Terrestrial Regional Ecosystem Exchange Simulator (Trees), which explicitly models soil and plant hydraulics, to study genotype‐specific acclimating behavior in cotton (*Gossypium hirsutum*) under water deficit conditions. Trees takes in half‐hourly meteorological data (e.g. photosynthetically active radiation (µmol photons m^−2^ s^−1^), precipitation (mm), and wind speed (m s^−1^)), and parameters that constrain rhizosphere and xylem hydraulics, photosynthesis and respiration, and carbon allocation processes (Mackay *et al*., 2015) and simulates output responses such as leaf and soil water potentials, stomatal conductance, and rhizosphere fluxes. While this model has been applied to woody species in natural and semi‐natural ecosystems under drought and heat stress (Mackay *et al*., 2015; McDowell *et al*., 2016, 2019; Johnson *et al*., 2018), it has yet to be tested against evaluations of crop plants under managed field conditions, a necessary step towards the use of hydraulics models in quantitative genetics studies of plant water deficit response.

In 2010–2012, a cotton drought experiment was carried out at the Maricopa Agricultural Center (MAC) in the Desert Southwest (Maricopa, AZ, USA) on a bi‐parental mapping population (Pauli *et al*., 2016). These lines were evaluated for stress‐adaptive traits under controlled water deficit using a field‐based, high‐throughput phenotyping platform. Quantitative trait loci, that is, genomic regions associated with traits of interest, were discovered to vary over the course of the season, and the correlation strengths between proximally‐sensed measures and agronomic traits were also time‐dependent. These previous results are evidence that whole plant drought responses are emergent properties arising from feedback of complex underlying interactions, including plant development, and provide strong motivation to examine them using a process‐based modeling approach. Here, we leveraged that experiment as a springboard for exploring cotton drought response via a biophysical process‐based model.

As a woody perennial species originating from the arid coastal regions in northern Yucatán (Mexico) (d'Eeckenbrugge & Lacape, 2014; Wendel *et al*., 2018) and now cultivated globally as an annual crop across different climates, *G. hirsutum* represented an exceptional study organism with which to investigate crop G × E under water deficit using Trees. Goals of this work were as follows: first, to characterize hydraulic traits of cotton across a small but unique set of germplasm; second, to apply Trees as a tool to retrospectively examine plant performance under drought; and third, to use observed soil variation along with genotype‐specific hydraulics in *in silico* experiments to investigate interactions with drought scenarios.

## Materials and Methods

### Soil variability

**Fig. 1:**
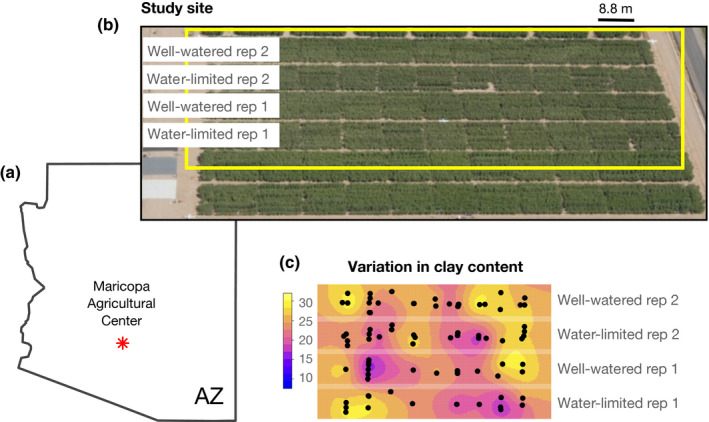
Experimental overview. (a) Map indicates location (red star) of the study site at Maricopa Agricultural Center within the state of Arizona, USA. (b) Aerial photograph showing the layout of the cotton drought experiment. (c) Plot showing variation in clay content (%) across the experimental treatments interpolated from 111 sampled field locations. Yellow indicates higher clay content and blue indicates lower clay content; black circles mark locations of soil sampling.

Previously unpublished data on soil characteristics from the 2010–2012 evaluations described in the Introduction section (and Supporting Information Methods S1) were analyzed in the current study. In 2010 and 2012, samples were collected using a tractor‐mounted soil boring machine (model 25‐TS; Giddings Machine Co., Windsor, CO, USA) at 56 and 55 locations, respectively, within the MAC field site (Maricopa, AZ, USA, lat. 33°04′37ʺN, long. 111°58′26ʺW, elevation 358 m asl) (Fig. 1). The locations of the neutron probes for 2011 matched those in 2010, and so sampling was not repeated. Samples were taken at each location from five depth intervals (0–30, 30–60, 60–90, 90–120 and 120–150 cm) and brought to the Agricultural Research Service (ARS) laboratory in Maricopa to determine soil particle size fraction (soil texture) using the Bouyoucos hydrometer method (Gee & Bauder, 1986) (Fig. S1). Soil samples collected in 2010 were also analyzed in the laboratory to determine the upper (field capacity) and lower (permanent wilting point) volumetric soil water contents at all incremental depths. Field capacity (FC) and permanent wilting point (PWP) soil water contents were determined at the −33 and −1500 kPa soil matric potentials, respectively, using pressure membrane extractors (Model 1000; Soilmoisture Equipment Corp., Santa Barbara, CA, USA) (Fig. S2). To derive values for plots from which samples had not been directly collected, values for the proportions of clay, sand, and silt were spatially interpolated using blocked kriging utilizing all 111 total soil sampling locations (2010 and 2012 combined) in the field to derive plot‐level estimates (see Methods S2). Data are provided in Table S1.

### Cotton hydraulic traits

At MAC in 2018, four visually‐contrasting genotypes with different breeding histories were selected for the analysis of stem and root hydraulic behavior out of 24 cotton (*Gossypium hirsutum* L.) accessions that were grown (24 accessions = 21 upland cotton accessions from the Gossypium Diversity Reference Set (GDRS) (Hinze *et al*., 2015) and three commercial cultivars). These genotypes were DP1549B2XF, Tipo Chaco, PD3 and VIR7094 Coker. DP1549B2XF (hereafter referred to as DP1549) is a modern commercial variety released in 2015 for the High Plains and Southwest regions of the USA; Tipo Chaco is a landrace collected from Trinidad and Tobago in 1947 during a collection expedition; PD3 is an improved variety developed in the USA with a Plant Variety Protection (PVP) issued in 1990 and targeted for production in the Eastern breeding region of the USA; and VIR7094 Coker (hereafter referred to as Coker) is a line originally developed as Coker 310 in the USA (PVP issued in 1974), shared with the Russian cotton breeding program, and then shared back with the USA germplasm collection. Plots were planted at a length of 4.5 m with the same interrow and alley spacing dimensions and management practices detailed in a previous study by Pauli *et al*. (2016). Irrigation was applied using a variable‐rate, overhead linear move irrigation system (Lindsay Corp., Omaha, NE, USA) with applications occurring across multiple days. Water‐limited (WL) and well‐watered (WW) conditions were implemented via irrigation scheduling, with two replications of genotypes per irrigation treatment with the WL treatment initiated at 50% flowering; scheduling of irrigation was performed on a weekly basis with model updates incorporating meteorological information.

Four whole plants, including the root system, per genotype were harvested on 5 September 2018 from the WW treatment. Root and shoot segments were excised under water and then placed in an ice bath within a cooler. Samples were shipped overnight on ice to the University of Utah, where vulnerability curves were constructed on three biological replicates per genotype (biological replicates = one stem and one root segment from three individual plants per genotype) using the centrifuge method (Alder *et al*., 1997). Hydraulic conductivity (*K*_h_; kg m s^−1^ MPa^−1^) was measured by flow onto a balance with a pressure head of 3–4 kPa (Sperry *et al*., 1988) and corrected for background flow (0 kPa pressure head; Hacke *et al*., 2000). Maximum conductivity (*K*_hmax_) was measured after emboli within samples were reversed by vacuum infiltration in KCl 10 mM solution for 1 h. Measurements were performed with degassed and filtered (0.2 µm) 10 mM KCl solution. Then samples were spun for 6 min at each pressure value before assessment of conductivity. Stems were spun using a rotor that fitted 14‐cm segments and roots using a rotor that fitted 10‐cm segments. Foam pads were placed in the water reservoirs to ensure stem and root ends were immersed in water when the rotor stopped (Tobin *et al*., 2013). All samples were processed within 3 d of sample collection. Data were reported as specific conductivity (*K*_s_, kg m^−1^ s^−1^ MPa^−1^), which was calculated by dividing *K*_h_ by the mean cross‐sectional area of the segment, and as a percent loss of conductivity (PLC, %) with respect to *K*_smax_ (i.e. values at 0 MPa in Table S2). Hydraulic vulnerability curves (VCs) were constructed by fitting measurements to a Weibull function:

(Eqn 1(a)) $$K_{s}=K_{s\;\max}\times e^{‐{\left(‐\psi /b\right)}^{c}}$$

(Eqn 1(b)) $$\text{PLC}=100\times (1‐e^{‐{\left(‐\psi /b\right)}^{c}})$$

where ψ is xylem pressure and *b* and *c* are scale (negative MPa) and shape (dimensionless) parameters fitted to the data, respectively (Sperry *et al*., 1998). Trees takes in the *b* and *c* parameters as inputs from Eqn 1(b).

DP1549 was selected for follow‐up evaluation in 2019 as it exhibited a mid‐range VC of the three improved varieties in 2018, and as a current commercial cultivar, was potentially the most representative of modern‐day breeding germplasm. Management of the 2019 MAC field site followed procedures from 2018. Construction of VCs was repeated using six biological replicates from the WW treatment and six biological replicates from the WL treatment (Fig. S3). Plant harvesting occurred on 10 September 2019 to match the developmental stage of samples collected from 2018, and harvesting, packing, shipping, and VC construction followed the same protocols described above. Vessel length distribution of stem xylem was determined using the silicon injection method (Sperry *et al*., 2005; Christman *et al*., 2009) on six additional biological replicates of DP1549 sourced from the same harvesting event as for VC sampling (Methods S3; Fig. S4). Water potential of transpiring and non‐transpiring leaves were measured on 11 September 2019 on another six WW replicates and six WL replicates of DP1549, which remained in the field. Briefly, pairs of leaves on the same branch were selected per plant in the morning at *c.* 11:00 h, during which one of the pair was chosen to be the ‘non‐transpiring’ leaf. This leaf was gently sealed in a Ziploc bag covered in aluminum foil to avoid transpiration and allow for equilibration with stem xylem pressure, while the other leaf remained uncovered and represented the ‘transpiring’ leaf. Between 13:25 and 15:09 h, leaves were excised and water potential was measured using a pressure chamber (3000 Series Plant Water Status Console; Soilmoisture Equipment Corp.).

### Trees simulations of cotton

The main parameters selected for modeling cotton are presented in Table 1. The *b* and *c* parameters from the PLC fit (Eqn 1b) were utilized directly to constrain the hydraulic sub‐model (Sperry *et al*., 1998) in Trees (Mackay *et al*., 2015). For soil inputs, plot‐level data were aggregated up to larger grid cells, each of which was comprised of 10 adjacent plots of experimental entries (Fig. S1). Per grid cell, Trees utilized soil texture characteristics as parameters and pedotransfer functions presented by Rawls *et al*. (1992) to derive soil hydraulic properties needed by the hydraulics sub‐model. Representative cells for validation modeling were selected using the following criteria: they were found either above the 90^th^ or below the 10^th^ percentiles for clay fractions, and they contained two or more directly sampled plots in the 2012 evaluation, thereby minimizing potential effects of interpolation uncertainty. Averaging a high clay grid cell from WW and a high clay grid cell from WL, a representative ‘high clay’ (HC; 27.8%) parameter set was derived. The same was repeated to derive a parameter set for the ‘low clay’ (LC; 19.0%) condition. The Trees input parameters of porosity and bulk density were estimated using the Soil Water Characteristics model implemented in Spaw, a USDA water budgeting tool (Saxton & Willey, 2005), by inputting soil texture values for the representative HC and LC conditions and assuming a soil organic matter content of 0.8%, as indicated by Post *et al*. (1988). Validation simulations were performed for HC and LC conditions for each irrigation regime, totaling four environmental scenarios: well‐watered, high clay (WW‐HC); well‐watered, low clay (WW‐LC); water‐limited, high clay (WL‐HC); and water‐limited, low clay (WL‐LC).

**Table 1:** Primary parameters for Trees cotton simulations.

Parameter type	Parameter name	Unit	Low clay	High clay	Source
Hydraulics properties	*G*_sref_	mol m^−2^ s^−1^	0.63	0.63	This study
	E at saturated hydraulic conductivity	mmol m^−2^ s^−1^	11.4	11.4	This study
	Predawn leaf water potential at saturated hydraulic conductivity	MPa	−0.8	−0.8	This study
	Midday leaf water potential at saturated hydraulic conductivity	MPa	−2.4	−2.4	This study
	Weibull *b* parameter, shoot	MPa	1.55	1.55	This study
	Weibull *c* parameter, shoot	Dimensionless	0.75	0.75	This study
	Weibull *b* parameter, root	MPa	0.29	0.29	This study
	Weibull *c* parameter, root	Dimensionless	0.59	0.59	This study
Soil properties	Silt fraction	Dimensionless	0.174	0.182	This study
	Clay fraction	Dimensionless	0.19	0.278	This study
	Soil bulk density	Mg m^−3^	1.59	1.56	This study
	Geometric standard deviation of soil particle size	mm	12.226	12.226	This study
	Geometric mean particle diameter	mm	0.1448	0.0764	This study
	Porosity	m^3^ m^−3^	0.401	0.4105	This study
Plant growth traits*	Specific leaf area	m^2^ leaf area kg^−1^ C	30.5	14.5	This study
	Root area to leaf area at saturated hydraulic conductivity	cm^2^ cm^−2^	2.5	2.5	Calibrated
	Leaf area index	m^2^ m^−2^	Varies	Varies	Literature^†^

* For parameterization of root distribution, see Supporting Information Fig. S6.

† Pauli *et al*. (2016).

For other cotton Trees parameters, because hydraulic data were not collected in the original study of Pauli *et al*. (2016), observations were made during a field evaluation during Summer 2019 on DP1549, PD3, Tipo Chaco, and VIR7094 Coker. Briefly, the field experiment planted in 2018 was repeated following the same methodology and experimental design, except with a different randomization of genotypes to experimental plots. During the crop's vegetative growth stage prior to flowering (22–29 July 2019), leaf water potential was measured during predawn (before 05:00 h) and midday (between 10:30 and 14:30 h) (3005 Plant Water Status Console; Soil Moisture, Goleta, CA, USA) along with leaf‐level gas exchange (LiCor 6400XT; Li‐Cor Biosciences, Lincoln, NE, USA) from the WW treatment. Cuvette settings were: flow rate, 300 μmol s^–1^; CO_2_, 400 μmol^–1^ mol air; photosynthetically active radiation (PAR), 2000 μmol photon m^–2^ s^–1^. These data were used to inform Trees input values found in Table 1 for transpiration, predawn/midday leaf water potentials and Gsref (stomatal conductance at vapor pressure deficit of 1kPa) (Oren *et al*., 1999). For the specific leaf area parameter, leaf area using LI‐3100C leaf area meter (Li‐Cor Biosciences) and dry weight were measured on 40 leaves harvested over the same days as gas exchange and leaf water potential were measured. Specific leaf area (m^2^ kg^−1^ dry weight) was converted to a per unit carbon basis assuming 38.6% carbon per unit dry weight for cotton leaves (Radin & Eidenbock, 1986).

Simulations began on day of year (DOY) 160 and ended on DOY 240. Forcing (i.e. meteorological) data for 2012 were taken from the Arizona Meteorological Network (AZMET) weather station by interpolating its hourly information into half‐hourly (i.e. moving average), except for total water input, which included precipitation from AZMET plus the measured applied irrigation from management records for both the well‐watered and water‐limited treatments (Fig. S5).

Processes underlying the dynamic responses of plant development to time‐varying environmental stress are less understood compared to responses that can be modeled primarily on first principles, for example, hydraulics. Canopy development in cotton typically follows a sigmoidal growth trajectory over the course of the season (Modala *et al*., 2015), so rather than having a fixed value, we set leaf area index (LAI) to increase in three to five stages within the range of observations made in the original study by Pauli *et al*., 2016. Roots were calibrated against available soil water content information by adjusting the proportion of root area relative to total root area, which is determined based on an inputted relationship to total leaf area (Sperry *et al*., 1998) (Fig. S6); that is, the root to shoot area ratio (a single value) was tuned up or down to generally capture overall water taken up across five root‐soil depths.

Observations from the 2012 study were used to corroborate Trees simulations. Soil water content was monitored approximately two times per week, and samples were taken from five comparable depths for soil texture characterization. A total of 19 time‐points coincided with the simulation period and therefore could be used for comparison to validate modeled soil water content. Canopy temperature, sensed by an Apogee SI‐121 infrared radiometer (IRT; Apogee Instruments, Logan, UT, USA) on the high‐throughput phenotyping platform, was used to evaluate modeled canopy temperature. Fourteen time‐points distributed across 6 d (DOY 201, 202, 208, 215, 216 and 222) coincided with the simulation period and were used for validation of temperature dynamics. Aggregated grid cell‐level values from plot‐level sensed data were used for comparison to match the modeling unit used in the simulations. Root mean square error (RMSE) of predicted vs observed data for volumetric soil water content was computed as follows:

(Eqn 2) $$\text{RMSE}=\sqrt{\frac{1}{N}\sum_{i=1}^{N}{\left(Y_{f_{i}}‐Y_{o_{i}}\right)}^{2}},$$

where $Y_{f_{i}}$ is the simulated value, and $Y_{o_{i}}$ is the grid cell‐level value aggregated from observed plots, and *N* is the total number of available modeled‐observation pairs. Hydraulic safety margin was computed as the difference between maximum transpiration potential (*E*_crit_) and actual (simulated) transpiration (*E*_c_) (Sperry *et al*., 2002).

### Genotype‐informed sensitivity analyses

**Fig. 2:**
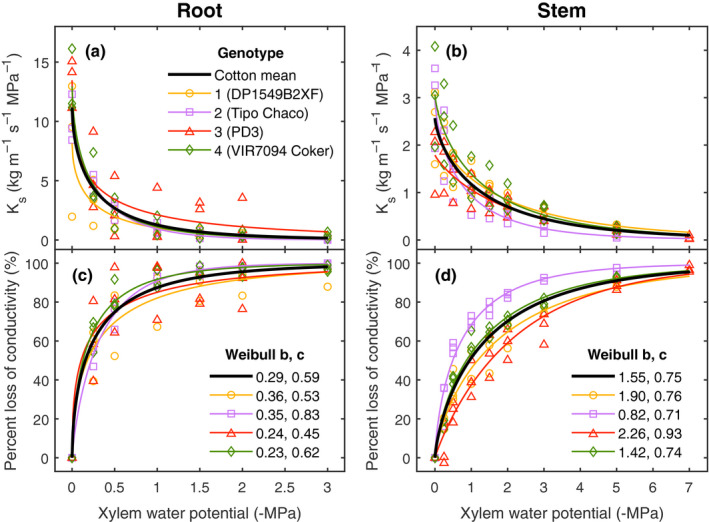
Cotton (*Gossypium hirsutum*) xylem vulnerability curves. Root (a, c) and stem (b, d) vulnerability curves showing xylem specific conductivity (*K*_s_) (a, b) and percent loss of conductivity (PLC) (c, d) vs xylem water potential. Curves were constructed with three root and three stem samples per genotype (DP1549B2XF, orange circles; Tipo Chaco, purple squares; PD3, red triangles; VIR7094 Coker, green diamonds). Solid curves represent the best fit Weibull function to each genotype (corresponding color) and to the mean of the species (all 12 samples, black lines). The root and stem Weibull *b* and *c* parameters used to parameterize the model are the ones from the PLC fits (c, d). Weibull *b* parameter was significantly different among genotypes for stems (*P* = 0.003) but not for roots (*P* > 0.05).

To explore model sensitivity to genotype‐specific vulnerability curves (VCs), simulations were performed using mean soil textural data averaged across all 44 grid cells. This soil parameter set was characterized by 24–58–18% of clay‐sand‐silt proportions, respectively. For simulation simplicity and to focus on the consequences of varying stem hydraulic VCs, we fixed LAI at 3.5 m^2^ leaf area m^−2^ ground area. The five VCs used were the four curves parameterized at the genotype‐level in addition to the overall parameterization (‘species’) (Fig. 2b,d). Simulations were performed under three scenarios (E1, E2 and E3) that differed in the severity of water limitation. E1 represented the least severe scenario, while E2 and E3 received 50% and 25%, respectively, of the water that E1 received during the drought period. All other meteorological drivers matched those used previously for the validation simulations. Percentage loss of whole plant hydraulic conductance (PLK) from simulation results was defined as follows:

(Eqn 3) $$\text{PLK}=100\times \left(1‐\frac{k_{\text{plant}}}{k_{\max}}\right),$$

where *k*_plant_ is whole plant hydraulic conductance (mmol H_2_O m^−2^ s^−1^ MPa^−1^) of the whole plant and *k*_max_ is the maximum (or saturated) whole plant hydraulic conductance (mmol H_2_O m^−2^ s^−1^ MPa^−1^) calculated as:

(Eqn 4) $$k_{\max}=\frac{E_{\text{sat}}}{\psi_{\text{pd}}‐\psi_{\text{md}}},$$

where *E*_sat_, ψ_pd_, and ψ_md_ are Trees parameters that describe transpiration rate (mmol H_2_O m^−2^ s^−1^), predawn leaf water potential (MPa) and midday leaf water potential (MPa) under saturated conditions, respectively. Saturated conditions in the model indicate conditions when the hydraulic pathway is fully charged. Relative hydraulic safety margin (ρ) (unitless) was defined as in Tai *et al*. (2017) and Johnson *et al*. (2018):

(Eqn 5) $$\rho =\frac{\left(E_{\text{crit}}‐E_{c}\right)}{\max(E_{\text{crit}}‐E_{c})}.$$

Vulnerability curves from DP1549 and Tipo Chaco, which gave rise to the two most contrasting behaviors in simulations with ‘average’ soil, were selected for follow‐up modeling to investigate performance under contrasting soil textures. Trees was used to simulate these genotypes under high clay and low clay conditions and the scenarios E1–E3, totaling 12 simulations (two genotypes × two soil conditions × three treatments). Relative difference for midday *E*_c_ was computed as follows:

(Eqn 6) $$\delta_{i}=\frac{\text{Tipo}\_E_{c\;i}‐\text{DP}\_E_{c\;i}}{\text{mean}(\text{Tipo}\_E_{c\;i},\text{DP}\_E_{c\;i})},$$

where Tipo_*E*_c_ *_i_* is midday *E*_c_ (mmol H_2_O m^−2^ s^−1^; averaged from 12:00 to 14:00 h) from the simulation parameterized by Tipo Chaco's vulnerability curve at day *i*, and DP_*E*_c_ *_i_* is midday *E*_c_ from the simulation parameterized by DP1549's vulnerability curve at day *i*. A positive value for δ*_i_* equates to the Tipo Chaco simulation having greater midday transpiration at day *i*, and a negative value indicates the DP1549 simulation being greater.

## Results

### Soil variability

Proportions of clay, sand, and silt averaged by depth at 56 locations in 2010 and 55 locations in 2012 were consistent with the MAC Casa Grande Series soil texture percentages reported by Post *et al*. (1988). Observed values spanned large ranges for all three soil mineral constituents (clay: 10.2–42.9%; sand: 36.4–78.4%; and silt: 4.7–30.7%) (Table S1) and interpolated soil textures showed spatial variation across both well‐watered and water‐limited treatments (Fig. 1). Clay had the highest coefficient of variation across grid cells compared to sand or silt (Table S3), and so it was used as the determinant to select contrasting cells, each composed of 10 plots (Table S4). Representative cell‐level soil textures were 28–54–18% clay‐sand‐silt (United States Department of Agriculture Natural Resources Conservation Service (USDA NRCS) sandy clay loam texture) for the high clay condition and 19–64–17% clay‐sand‐silt (USDA NRCS sandy loam texture) for the low clay condition.

### Cotton hydraulic traits

All samples displayed R‐shaped hydraulic vulnerability curves with a rapid decline in conductivity at high water potentials, followed by a long tail and much slower decline at low water potentials (Fig. 2). Pressure at 50% loss of conductivity (P50) for stem and root were −0.95 and −0.16 MPa, respectively. In absolute units, maximum specific conductivity was *c*. 4.5‐fold higher in roots than for stems at an overall average of 11.14 vs 2.55 kg m^−1^ s^−1^ MPa^−1^ in stems. Stems at −2 MPa retained conductivities of 0.70 kg m^−1^ s^−1^ MPa^−1^, while roots at −2 MPa pressure retained hydraulic conductivities of 0.40 kg m^−1^ s^−1^ MPa^−1^. A xylem pressure of −2 MPa is within the range values observed for non‐transpiring leaves around midday in 2019. Both of these conductivity values were above the *K*_s_ threshold below which drought‐induced mortality risk significantly increases for some shrub species, which is at 0.20 kg m^−1^ s^−1^ MPa^−1^ (Venturas *et al*., 2016). There were no significant differences in root VC parameters across genotypes (*P *> 0.05 for each parameter; one‐way analysis of variance), although there were for genotype‐specific curves of stems; we report significant differences in the *b* parameter and P50 for stem curves (*P* = 0.003 and 0.007, respectively). Stems of PD3 had the most negative P50 value (−1.52 MPa), while stems of Tipo Chaco had the least negative (−0.49 MPa); these two genotypes were significantly different for both *b* and P50 (*P* < 0.05; *post‐hoc* Tukey test). Vulnerability curves in stems showed high agreement when measured during a second season (Fig. S3), and no differences were detected between curves of samples taken from the WW vs WL treatment in our experiments where deficit watering was initiated at flowering. Stem median and mean xylem vessel length (± SD) were 7.1 ± 1.6 cm and 9.1 ± 1.3 cm, respectively (*n* = 6; Fig. S4).

### Trees simulations of cotton

**Fig. 3:**
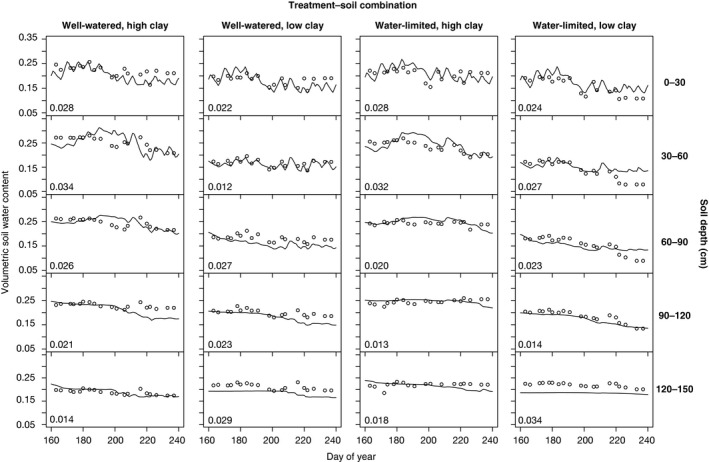
Trees model validation. Validation results from species‐level 80‐d simulations performed at grid‐level for four scenarios: well‐watered, high clay; well‐watered, low clay; water‐limited, high clay; water‐limited, low clay. Data on volumetric soil water content were used to validate modeled rhizosphere volumetric soil water content across five 30 cm soil layers. Circles are observed values and lines are simulations. Root mean square errors per soil layer are shown in plots.

To assess the suitability of Trees for application to cotton drought experiments, we conducted validation simulations and compared modeled outcomes to observations from the 2012 evaluation, as that year yielded the richest empirical dataset (Pauli *et al*., 2016). Estimates of LAI, computed from normalized difference vegetation index (NDVI) and plant height sensed by the high‐throughput phenotyping platform, were provided by Pauli *et al*. (2016) for the experimental period after drought initiation. Modeled soil water content was able to capture the general water content dynamics observed across five soil depths, with depth‐specific RMSE ranging from 0.013 to 0.034 (Fig. 3). Observed canopy temperature values sensed by the high‐throughput phenotyping platform were compared to modeled canopy temperatures, and we found that WL‐LC simulations matched observed values the closest, while WL‐HC simulations showed the greatest deviations from observations (Fig. S7).

Simulation results from model validation assisted retrospective examination of physiological traits that were not measured. Plant hydraulic safety differed between the four simulations, with the high clay simulations typically having less safety margin, that is, the difference between maximum and actual transpiration, as compared with the low clay simulations prior to the initiation of drought. The high clay content led to lower water matric soil potentials for the same soil water content compared to lower clay texture (see also Fig. S2). However, the changes in water potential are more progressive and predictable than with coarser soils, thus, the plants may extract more water (i.e. exhibit actual transpiration closer to maximum transpiration) than in a coarse soil where there is a point at which small variations in volumetric soil water content lead to large changes in water potential, which could lead to complete hydraulic failure. Greater differences in leaf water potential extremes were seen in high clay simulations between the well‐watered and water‐limited simulations, and there were similar patterns for transpiration and stomatal conductance. Since these results could potentially be attributed to LAI rankings, where differences in LAI were greater in high clay than low clay between the two water treatments (as directly informed by observed LAI), a series of *in silico* experiments were conducted that allowed us to directly interrogate the effects of variables of interest (hydraulic vulnerability curves, soil textures, and water inputs) on plant performance.

### Genotype‐informed sensitivity analyses

**Fig. 4:**
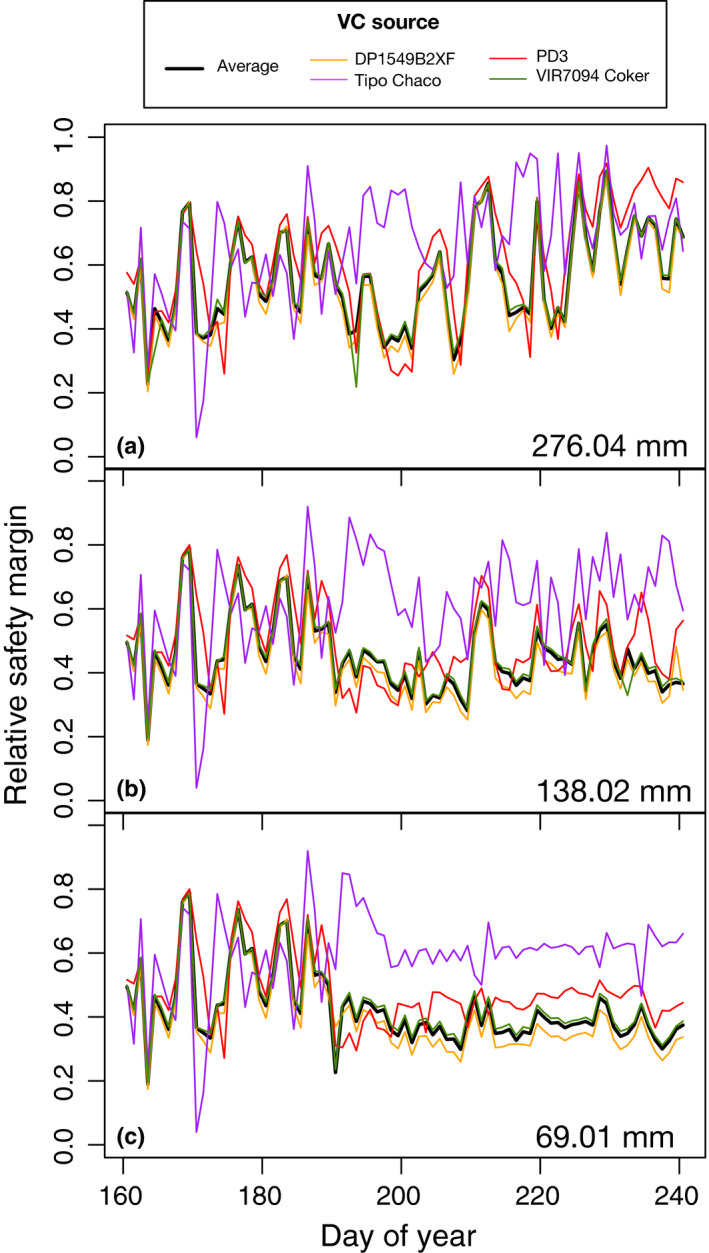
Sensitivity of relative safety margin to genotype‐informed hydraulic vulnerabilities under three water input scenarios. Model results of sensitivity analysis using five hydraulic vulnerability curves: four were derived from genotype‐specific data and one was estimated using combined data of all genotypes. Models were run under three conditions that varied in the severity of water limitation from least stress (E1) (a) to most stress (E3) (c). Plotted here are daily values at around midday (12:30 h). Water input becomes variable across the three scenarios starting on day of year 188 (arrow) and the sum of water inputs after day 188 in each scenario is indicated within the panels.

We investigated the consequences of varying stem VCs, informed directly by genotype‐level and overall VC parameters (five total sets) (see the Materials and Methods section). Model results under E1, the scenario of least water deficit, demonstrated a strong differentiation between Tipo Chaco simulations (i.e. simulations informed by the VC of Tipo Chaco) and all others with respect to soil water potential and leaf water potentials (Figs S8, S9). Analyzing distributions of ψ_leaf_ at predawn (03:00–05:00 h average) and approximate midday (12:00–14:00 h average) supported the idea that Tipo Chaco simulations behaved uniquely. Although all the *in silico* genotypes displayed acclimating behavior of stomatal closure under increasing water stress from E1 to E2 with increased leaf water potentials around midday, Tipo Chaco was the least affected, in relative terms, such that E2 simulations resulted in convergent behavior across the genotypes for approximate leaf water potentials. Conversely, simulations with the other vulnerability curves were least affected, in relative terms, from E2 to E3; this resulted in E3 simulations showing divergent behavior between Tipo Chaco and all others, which each exhibited a skewed distribution with individual days of low midday pressure (< −2 MPa). Under the scenario of least water deficit (E1), no differences were observed in relative safety margin (ρ) between the genotypes (Fig. 4). This is consistent with the supposition that under scenarios without much water stress, plants are performing close to their individual maximum and thus genotypic differences may not be expected for relativized measures of safety. Increasing drought stress in scenario E2 resulted in Tipo Chaco expressing a higher relative safety margin, while all others performed similarly to each other and displayed lower relative safety margins. Under the most severe scenario, with just 69.01 mm of water input during the drought period, genotypes could be separated by relative safety margin and DP1549 displayed the lowest relative safety margin, indicating that it was able to draw more water from the soil (Fig. S8) relative to the other *in silico* genotypes.

We asked whether the extent of soil textural variation observed on the MAC farm would give rise to soil by genotype interaction under drought, given that genotypic rankings are fundamental for genetic mapping. *In silico* genotypes Tipo Chaco and DP1549 showed the most contrasting relative safety margins under the greatest water deficit and were thus chosen for further model runs (Fig. 4). Relative safety margin was used for the selection because this metric had previously been shown to best explain plant mortality response to water deficit in naturally‐occurring environments compared to other modeled or directly observed metrics such as soil water content, sensitivity of stomatal closure to leaf water potential, differences between leaf water potential and P50, hydraulic safety margin (also called water use envelope), and sensitivity to vapor pressure deficit (Tai *et al*., 2017; Johnson *et al*., 2018). Simulations in E1–E3 were run under high clay and low clay conditions (two VCs × three environmental (water input) scenarios × two soil conditions = 12 simulations). Leaf water potentials in general were lower in high clay scenarios than low clay, with DP1549 simulations dropping lower than Tipo Chaco around midday in nearly all cases (Fig. S10). Under well‐watered conditions prior to day 188 when drought was initiated in all three scenarios, transpiration in DP1549 was higher than Tipo Chaco, and these differences were shown to be consistently greater under conditions of low clay. In E1, with the least water stress, DP1549 consistently exhibited higher transpiration than Tipo Chaco, except for two notable instances where Tipo Chaco had higher transpiration rates under the high clay condition. These events were preceded by water deficit periods lasting several days (Fig. S5).

**Fig. 5:**
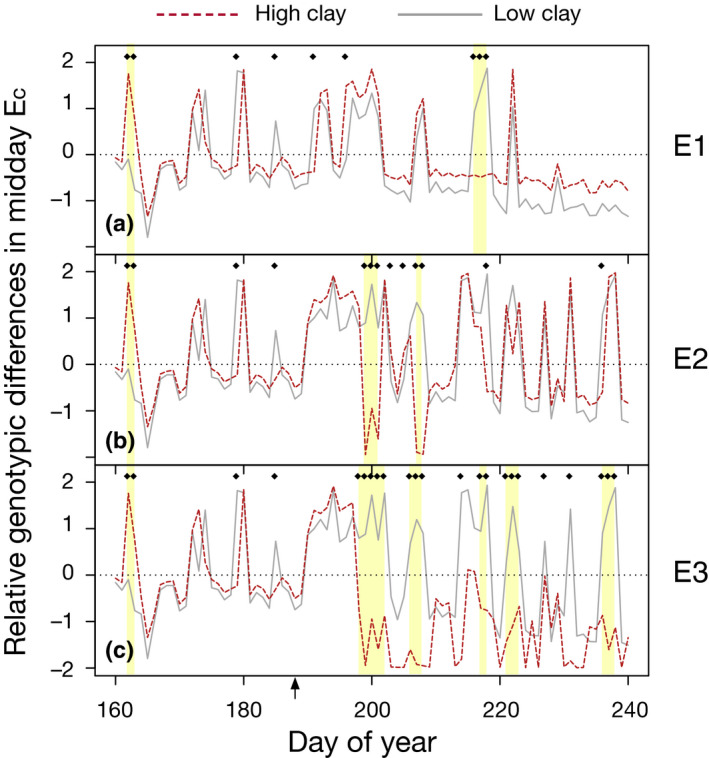
Soil by genotype interaction for midday transpiration rates under three water input scenarios. Relative transpiration differences, taken around midday (12:00–14:00 h daily averages), between simulations informed by DP1549's vulnerability curve and simulations informed by Tipo Chaco's vulnerability curve (VC) under high clay (maroon dashed line) and low clay conditions (grey lines). Three environmental scenarios were applied that differ only in water inputs after day of year 188 (arrow), with the most water in E1 (a) to the least water in E3 (c). Positive values indicate that simulations parameterized by Tipo Chaco's VC had greater values of transpiration, while negative values indicate the opposite genotypic ranking. Black diamonds mark days that showed a divergence with respect to the two soil conditions (e.g. high clay is negative and low clay is positive), which represent days where genotype ranking flips with respect to soil. Yellow shaded boxes indicate time windows in which there are two or more consecutive days of divergence of the same direction.

Relative genotypic differences were more pronounced under scenarios of greater stress, E2 and E3 (evident as larger amplitudes in Fig. 5), and also displayed instances of soil by genotype interaction, where genotypic ranking depended upon soil condition. While soil by genotype interactions emerged under all three environmental scenarios, they were especially frequent under E3 (Fig. 5). This result suggested that under scenarios without much water stress, differences across individuals may be attributed primarily to their inherent genotypic differences (parameterized here by VCs only) while under greater water stress, environmental factors come increasingly into play to give rise to complex interactions. Under very severe stress, when individuals will have closed their stomata, more consistent genotypic rankings may be recovered again.

## Discussion

In this study we used a process‐based model to evaluate the consequences of hydraulic traits, soil textures, and water inputs on plant drought response. Leaf water potential and transpiration results of Tipo Chaco simulations were consistent with this genotype having the steepest stem hydraulic vulnerability curve; these simulated plants exhibited a ‘conservative’ behavior under water deficit and had less water uptake from the soil over the course of all drought scenarios as compared to the other genotypes. Less water uptake resulted in greater soil water potentials over the course of drought and allowed Tipo Chaco to maintain the highest values of relative safety margin, especially under increasing drought severity. Traits that confer conservative hydraulic behavior have been suggested to support greater agricultural productivity under mild or moderate water deficit (Messina *et al*., 2015; Sinclair *et al*., 2017). However, low genotypic stomatal conductance and transpiration traits with water‐saving benefits should also be assessed for potential trade‐offs, especially under scenarios where drought co‐occurs with high temperature as transpiration serves to cool canopies and mitigate heat stress. Future detailed genotype‐level parameterization of cotton with respect to carbon acquisition and plant development (including characterization of sink strength) will be needed in order to investigate these trade‐offs using a process‐based modeling framework.

Connecting genetic variation to process‐based plant models for improved prediction under novel environments and greater understanding of mechanisms underlying differential genotypic performance has long been a sought‐after goal. Indeed, significant methodological efforts have been made in the last 15 yr, including the use of genetics‐ and genomics‐based models for parameterization (White & Hoogenboom, 1996; Yin *et al*., 1999; Wang *et al*., 2019), estimating genotype‐specific parameters using process‐based models (Lamsal *et al*., 2018), and wrapping process‐based models within genomic prediction frameworks to test for enhanced forecasting capabilities (Technow *et al*., 2015; Cooper *et al*., 2016). Our study adds to this body of work by exploring explicitly modeled hydraulics in the context of existing intra‐specific genetic variation. While this level of detail is unlikely to be necessary for prediction under well‐watered environments, our results showed that hydraulics‐driven genotype‐by‐environment interaction is prominent under water deficit, even when *in silico* genotypes are differentiated only by VCs. To enhance utility, models require a thoughtful balance between reality and parsimony that is dependent upon model application (Hammer *et al*., 2019); we suggest that for the purpose of examining consequences of genetic variation underlying dynamic physiological drought responses, widely‐used crop modeling platforms may benefit from additional complexity that considers hydraulic fluxes, conductances, and water potentials such as the approaches found in Trees or other models, for example, the modified Tardieu‐Davies model (Tardieu *et al*., 2015). On the other hand, one clear path for future Trees development for use in cotton is to integrate a dedicated crop growth routine, such as one from a cotton crop model (Thorp *et al*., 2017). This improvement would help to better capture the energy budget and will be necessary for modeling processes that lead to eventual yield (fiber) formation.

As a perennial species cultivated as an annual, cotton offered a unique modeling opportunity to link widely studied drought physiology of long‐lived woody species with rapid stress dynamics that annual crops can experience over the course of a single growing season. While only four genotypes were assessed for VCs in our study, representing a mere fraction of cotton's genetic diversity, we found statistically significant differences among the stem VCs, providing evidence that there is intraspecific genetic variation for this physiological trait. In contrast to the stem VCs, we were unable to detect evidence for genotypic differences in root VCs. This could be due to (1) a reduction of genetic variation in cotton root VCs due to natural selection optimizing this trait, given its significance to plant fitness, (2) a need for larger replicate size to account for root phenotypic plasticity, and/or (3) a need to sample more diverse genetic material. Future efforts to characterize root hydraulic vulnerability in cotton should therefore aim to address (2) and (3) in order to confirm or dispute (1).

The VCs in this study were R‐shaped and differ from those published by Li *et al*. (2020), who report roots and stem P50s of *c*. −5.5 MPa. It has been suggested that R‐shaped VCs are due to an open‐vessel artifact, but we ruled this out, demonstrating that only 5.7% of vessels were open through 14‐cm stem segments (Fig. S4). Moreover, the centrifuge methodology used in this study has been thoroughly tested for long‐vessel species with no support for an open‐vessel artifact (e.g. Sperry *et al*., 2012; Hacke *et al*., 2015). Thus, the discrepancy is hypothesized to be a result of one or both of the following: differences among plant materials: Li *et al*. (2020) used plants from a different genotype that was smaller in size and grown in pots under greenhouse conditions; differences in VC methodology: Li *et al*. (2020) VCs were constructed with the optical visual method. The optical method is not capable of capturing cavitation events that occur at water potentials higher than those already experienced by the plants, as emboli cannot first be reversed prior to VC construction. Additionally, direct comparison among methods highlight the fact that it is very difficult to estimate the loss of hydraulic conductance from optical techniques (Venturas *et al*., 2019; Pratt *et al*., 2020). Nevertheless, despite the difference in VCs, what is interesting is that both our simulations and 2019 field measurements show that stomata are at near‐complete closure when leaf water potentials drop below *c*. −2.5 MPa, in agreement with the range reported by Li *et al*. (2020).

It is noteworthy that accessions DP1549, PD3 and VIR7094 Coker were most similar in their curves while Tipo Chaco stood apart. DP1549, PD3, and VIR7094 Coker are improved varieties released by US breeding programs during different points in history, with DP1549 being the most modern (see the Materials and Methods section for more detailed descriptions), while Tipo Chaco is a landrace accession collected from a plant expedition trip to Trinidad and Tobago in the 1940s. It is interesting that Tipo Chaco exhibited the steepest vulnerability curve compared to the three improved varieties, as this characteristic is often interpreted as ‘more vulnerable’. We can speculate several reasons for this: first from the modeling results, we observe that this steeper curve confers an *in silico* conservative behavior under drought, and hence it may be adaptive for conditions where precipitation is not dependable; second, the other three improved varieties, which were actively bred for productivity, may have been indirectly selected for more resistant xylem to support higher levels of transpiration and carbon acquisition; and/or third, Tipo Chaco behaves differentially with respect to other processes that were not characterized in this study, such as photosynthetic capacity, which may decrease its demand for water as compared to the improved varieties. Given the difference in Tipo Chaco's vulnerability curve and its subsequent effects on modeled whole plant behavior, it would be of interest to sample *G. hirsutum* across its landrace groups for hydraulic traits in order to investigate the relationship between these traits and ecological niches, to understand the partitioning of variation within and among genetic groups, and to use process‐based modeling as one approach of testing trait potential for de novo crop domestication and improvement.

As regions of the world progress towards complete departure from historic climate variability (Mora *et al*., 2013), increased genetic and physiological understanding of both plant adaptation and acclimation to abiotic stress will be key to advancing robust tools that can guide varietal development and breeding under sub‐optimal conditions such as drought. Processed‐based models developed from current knowledge of plant physiology have the potential to support decision‐making, from designing field experiments to informing selection criteria and strategies. By making these types of tools accessible to plant breeders and geneticists, it is hopeful that more rapid genetic improvement can be achieved in the future, with the aim of developing crops with greater yield, enhanced climate resiliency, and decreased environmental impact.

## Author contributions

DRW, DSM and DP conceptualized the study, MAG, DH and DP led experiments at the MAC farm, KT managed model‐informed irrigation schemes, and MV led hydraulic trait characterization. DRW carried out Trees modeling and DSM supervised modeling efforts. DRW, MV and DP analyzed data. DRW wrote the manuscript, and all authors contributed writing and/or feedback.

## Supporting information


**Fig. S1** Soil variation in the cotton drought experiment at Maricopa Agricultural Center.
**Fig. S2** Pressure extraction results of soil samples collected in 2010.
**Fig. S3** Comparison of vulnerability curves for a 2‐yr period.
**Fig. S4** Measurement of xylem length distribution.
**Fig. S5** Environmental drivers.
**Fig. S6** Root parameterization.
**Fig. S7** Modeled and observed canopy temperature.
**Fig. S8** Sensitivity of soil water potential to varying hydraulic vulnerability curves under three water input scenarios.
**Fig. S9** Sensitivity of modeled leaf water potential to genotype‐specific hydraulic vulnerability curve under three water input scenarios.
**Fig. S10** Modeled leaf water potential response to contrasting genotype‐specific hydraulic vulnerability curves and soil textures.
**Methods S1** Experimental details at Maricopa Agricultural Center (MAC) in 2010–2012.
**Methods S2** Soil interpolation.
**Methods S3** Determination of xylem vessel length distribution.

**Table S1** Soil observations (years 2010 and 2012).
**Table S2** Observations from vulnerability curve construction in 2018.
**Table S3** Summary statistics for original soil texture variables.
**Table S4** Soil textures aggregated across cells.
Please note: Wiley Blackwell are not responsible for the content or functionality of any Supporting Information supplied by the authors. Any queries (other than missing material) should be directed to the *New Phytologist* Central Office.
