# Supplementary material for: Use of hydraulic traits for modeling genotype‐specific acclimation in cotton under drought
Source: New Phytol. 2020 Jul 18;228(3):898–909. doi: 10.1111/nph.16751 (PMC7586954; doi:10.1111/nph.16751)
Supplement: Supplementary file 1 — Fig. S1 Soil variation in the cotton drought experiment at Maricopa Agricultural Center. Fig. S2 Pressure extraction results of soil samples collected in 2010. Fig. S3 Comparison of vulnerability curves for a 2‐yr period. Fig. S4 Measurement of xylem length distribution. Fig. S5 Environmental drivers. Fig. S6 Root parameterization. Fig. S7 Modeled and observed canopy temperature. Fig. S8 Sensitivity of soil water potential to varying hydraulic vulnerability curves under three water input scenarios. Fig. S9 Sensitivity of modeled leaf water potential to genotype‐specific hydraulic vulnerability curve under three water input scenarios. Fig. S10 Modeled leaf water potential response to contrasting genotype‐specific hydraulic vulnerability curves and soil textures. Methods S1 Experimental details at Maricopa Agricultural Center (MAC) in 2010–2012. Methods S2 Soil interpolation. Methods S3 Determination of xylem vessel length distribution. [file NPH-228-898-s001.pdf]

***New Phytologist* Supporting Information**

Article title: Use of hydraulic traits for modeling genotype-specific acclimation in cotton under drought
Authors: Diane R. Wang, Martin D. Venturas, D. Scott Mackay, Douglas J. Hunsaker, Kelly R. Thorp, Michael A. Gore, Duke Pauli
Article acceptance date: 07 June 2020

The following Supporting Information is available for this article:

Fig. S1 Soil variation in the cotton drought experiment at Maricopa Agricultural Center

Fig. S2 Pressure extraction results of soil samples collected in 2010

Fig. S3 Comparison of vulnerability curves from two years

Fig. S4 Measurement of xylem length distribution

Fig. S5 Environmental drivers

Fig. S6 Root parameterization

Fig. S7 Modeled and observed canopy temperature

Fig. S8 Sensitivity of soil water potential to varying hydraulic vulnerability curves under three water input scenarios

Fig. S9 Sensitivity of modeled leaf water potential to genotype-specific hydraulic vulnerability curve under three water input scenarios

Fig. S10 Modeled leaf water potential response to contrasting genotype-specific hydraulic vulnerability curves and soil textures.

Table S1 Soil observations (years 2010 and 2012)

Table S2 Observations from vulnerability curve construction in 2018

Table S3 Summary statistics of original soil texture variables.

Table S4 Soil textures aggregated across cells.

Methods S1 Experimental details at Maricopa Agricultural Center (MAC) in 2010-2012

Methods S2 Soil interpolation

Methods S3 Determination of xylem vessel length distribution

Fig. S1 Soil variation in the cotton drought experiment at Maricopa Agricultural Center. (a) Field maps showing soil sample locations from 2010 and 2012 (black dots) and interpolated clay and sand content using combined soil textural data collected in 2010 and 2012. Cells, each comprising 10 plots, are demarcated with black borders. Selected high clay and low cells used to inform soil texture parameters used for modeling are marked white crosses (X) and white asterisks (*), respectively. **(b, c, d)** Distribution of cell-level (n=44 cell) soil characteristics averaged from 10 interpolated plot values per cell.

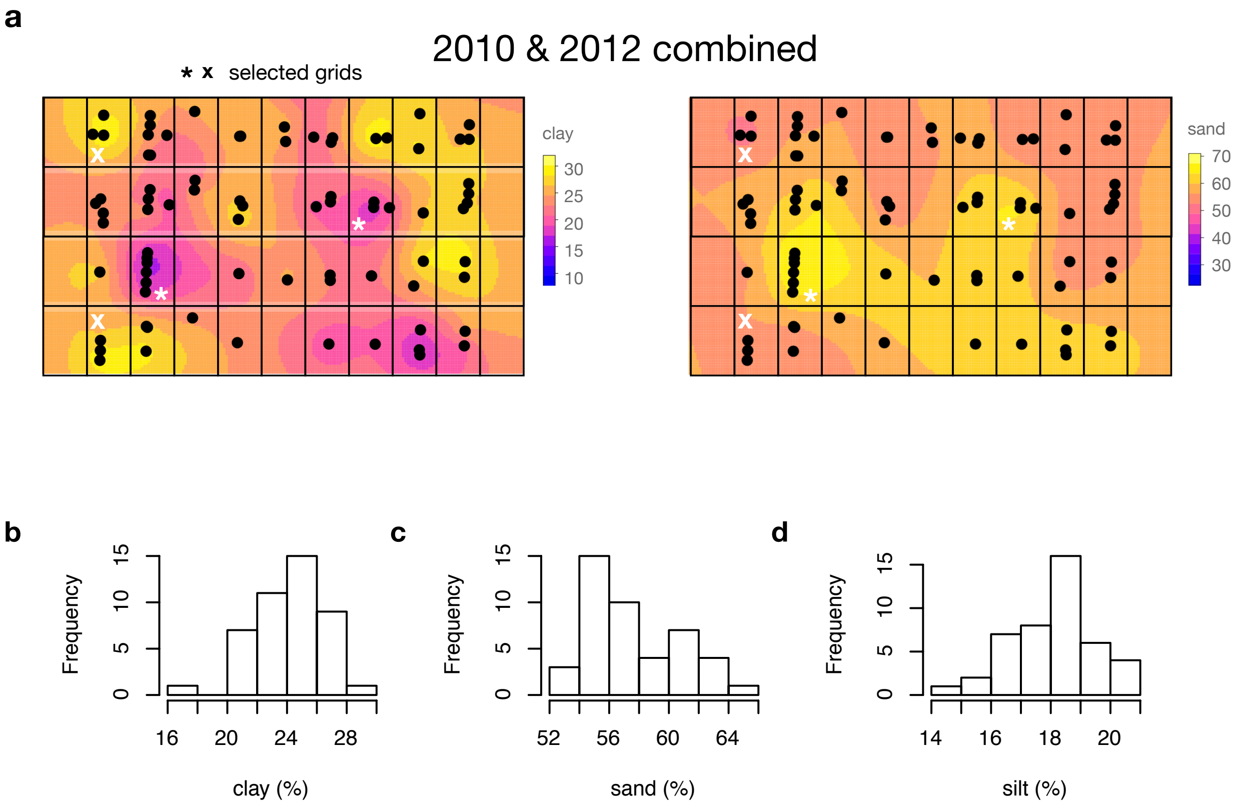

Fig. S2 Pressure extraction results of soil samples collected in 2010. The 2010 soil samples were analyzed in the laboratory to determine the upper (field capacity) and lower (permanent wilting point) volumetric soil water content (SWC; %) at incremental depths: 30, 60, 90, 120, and 150 cm. Volumetric SWC was computed by multiplying measured gravimetric SWC by average bulk density (1.574 g/cm^3). **(a)** Relationship between soil matric potential and volumetric SWC on sampled locations **(Figure S1c left panel)** averaged across depths per location. Black points show the overall mean volumetric SWC at each pressure. **(b-d)** Relationships between soil texture as depicted by clay content (%) and volumetric SWC on all collected samples (*i.e.,* not averaged across depth) at **(b)** -33 kPa (field capacity), **(c)** -500 kPa, and **(d)** -1500 kPa (permanent wilting point). A positive relationship is observed between clay content and volumetric SWC at each matric potential level. Examples of a high clay sample and a low sample are annotated in red and blue points, respectively. For this high clay sample (red) to exhibit a matric potential of -500 kPa, volumetric SWC would equal 22.51%. In contrast, for the low clay sample (blue) to exhibit the same matric potential, it would need to dry down to 8.66% volumetric SWC. This example illustrates how plants under drought that are growing in more clayey soils experience more stress than plants growing in sandier soils of equivalent SWC, due to needing to drop their leaf water potentials much further to extract water out of the soil and into the plant. Note under agricultural field settings, sandy and clayey soils very rarely have "equivalent SWC" and the sandy soils are the ones that exhibit stress sooner and yield more poorly.

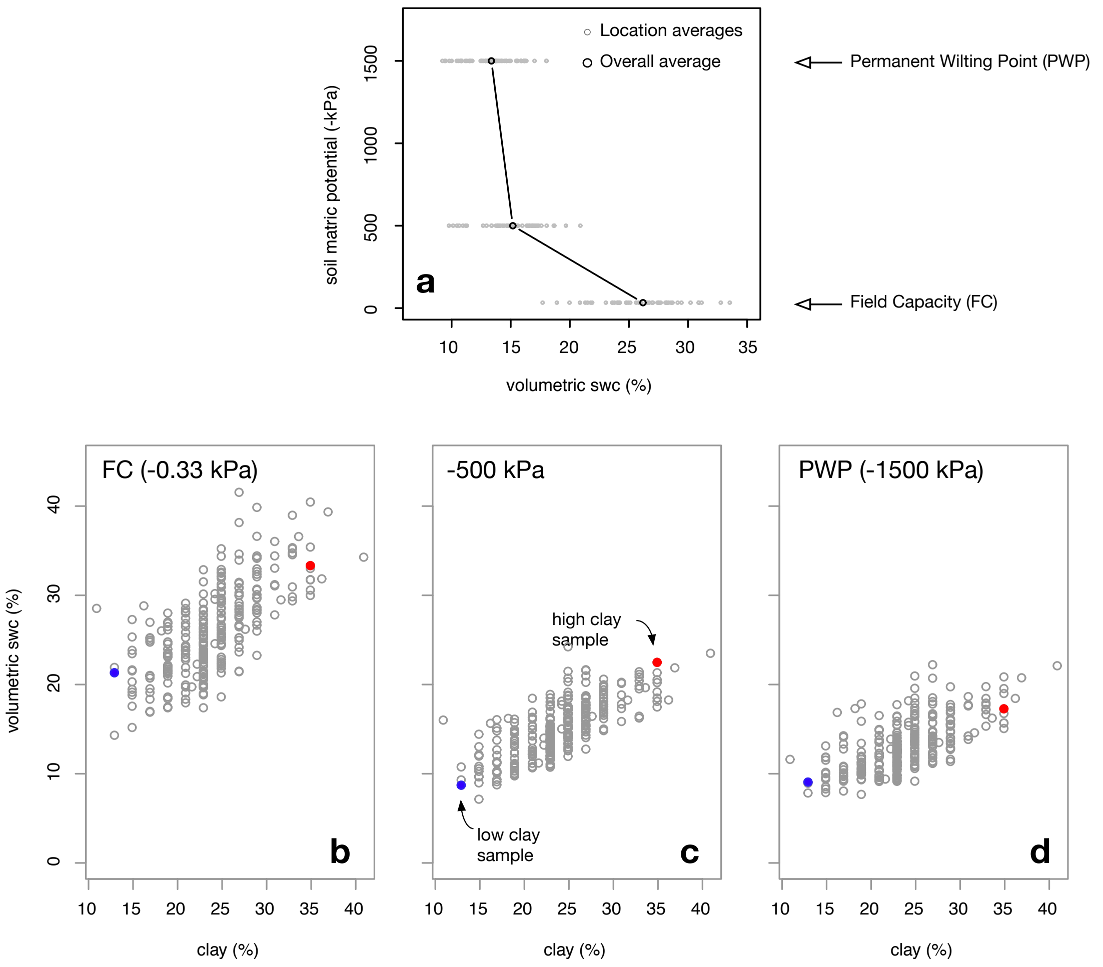

Fig. S3 Comparison of vulnerability curves from two years and watering treatments. We constructed vulnerability curves for stems of DP1549B2XF in September 2019. We compared curves from 2019 well-watered (purple squares, n=6) and 2019 water limited (red triangles, n=6) treatments, to those from 2018 stems (green circles, n=3, same samples as those presented in Fig. 2). **(a)** Specific conductivity (Ks) and **(b)** percent loss of conductivity vulnerability curves showed no differences among years and treatments. Solid lines of the corresponding colors show the best fit Weibull curves for each treatment and year.

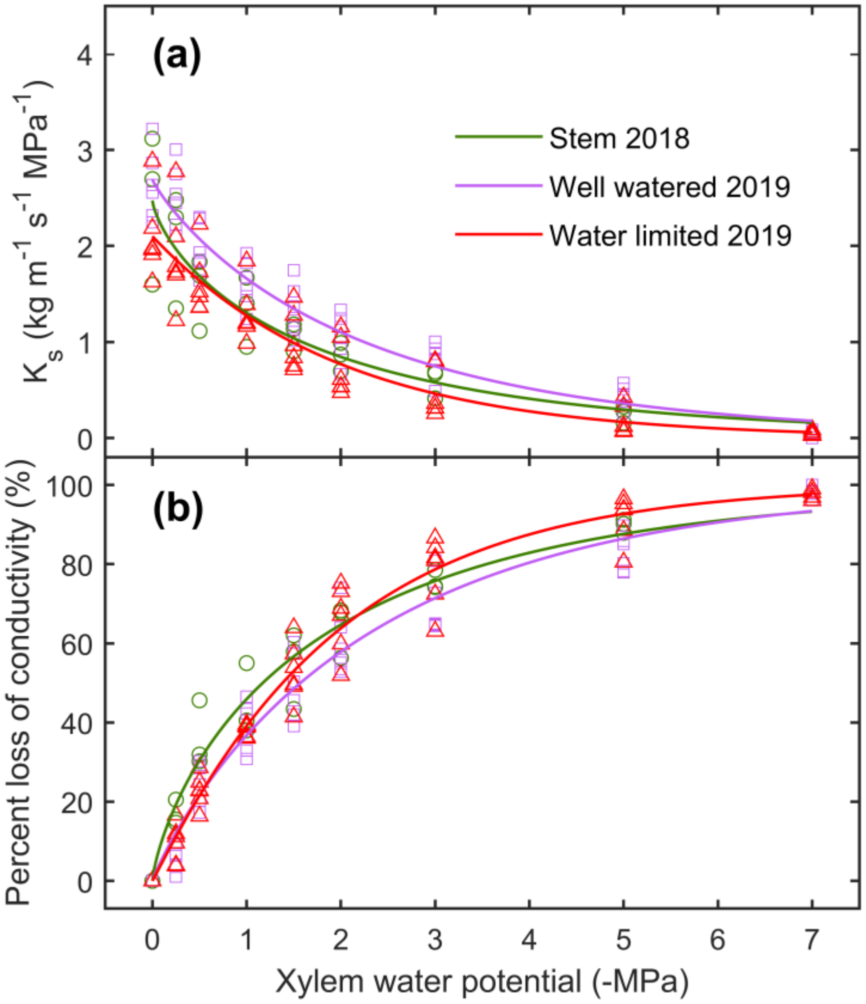

Fig. S4 Vessel length distribution of cotton stems. Representative cotton (*Gossypium hirsutum*) sample cross-section at 0 cm **(a, b)** and 14 cm **(c, d)** from the silicone injection point. Left panels show light transmittance images **(a, c)** and right panels are their corresponding ultraviolet (UV) fluorescence images **(b, d)**. Vessels that are filled with silicone (open to the injection point) glow under UV light (bright blue in **b, d**), whereas vessels that are not silicone filled do not glow (black in **d**). Only 5.1 % of vessels that were open at the injection site (0 cm) were open at 14 cm in this sample (bright blue in **d**). **(e)** The number of silicone filled vessels at different distances from the injection point was measured on six samples (different symbols) from genotype DP1549B2XF and a Weibull curve (black line) was fitted to all the data. **(f)** The proportion of vessels of 30 vessel length classes distributed logarithmically (black diamond) was calculated from the Weibull curve using the procedure described by Christman et al. (2009).

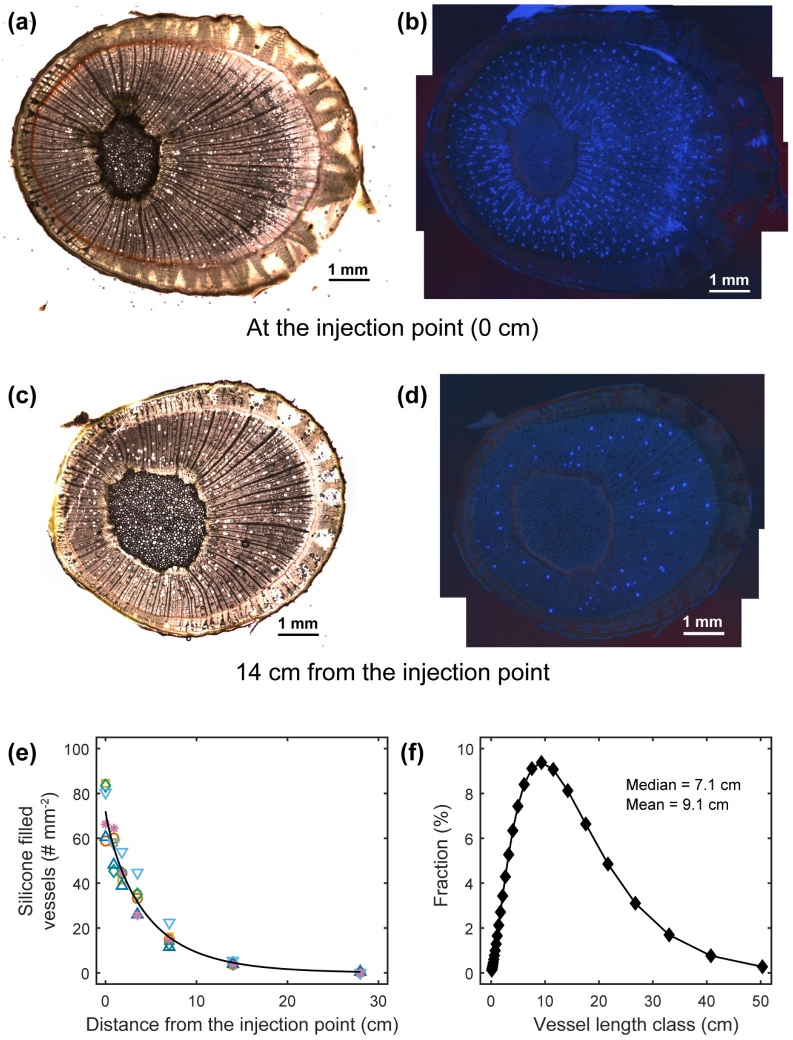

REFERENCE

Christman MA, Sperry JS, Adler FR. **2009**. Testing the 'rare pit'hypothesis for xylem cavitation resistance in three species of Acer. *New Phytologist* **182**: 664–674.

Fig. S5 Environmental drivers. Half-hourly meteorological information used by TREES modeling of the 2012 evaluation in Maricopa Agricultural Center. Shown here are air temperature (°C), solar radiation (Photosynthetically Active Radiation [PAR]; µmol m^{-2} s^{-1}), atmospheric vapor pressure deficit (VPD; kPa) and water inputs (precipitation + irrigation; mm) from the well-watered treatment. Water-limited treatment received one-half of the irrigation relative to the well-watered treatment after drought was initiated at day 188 shown by the vertical dotted line.

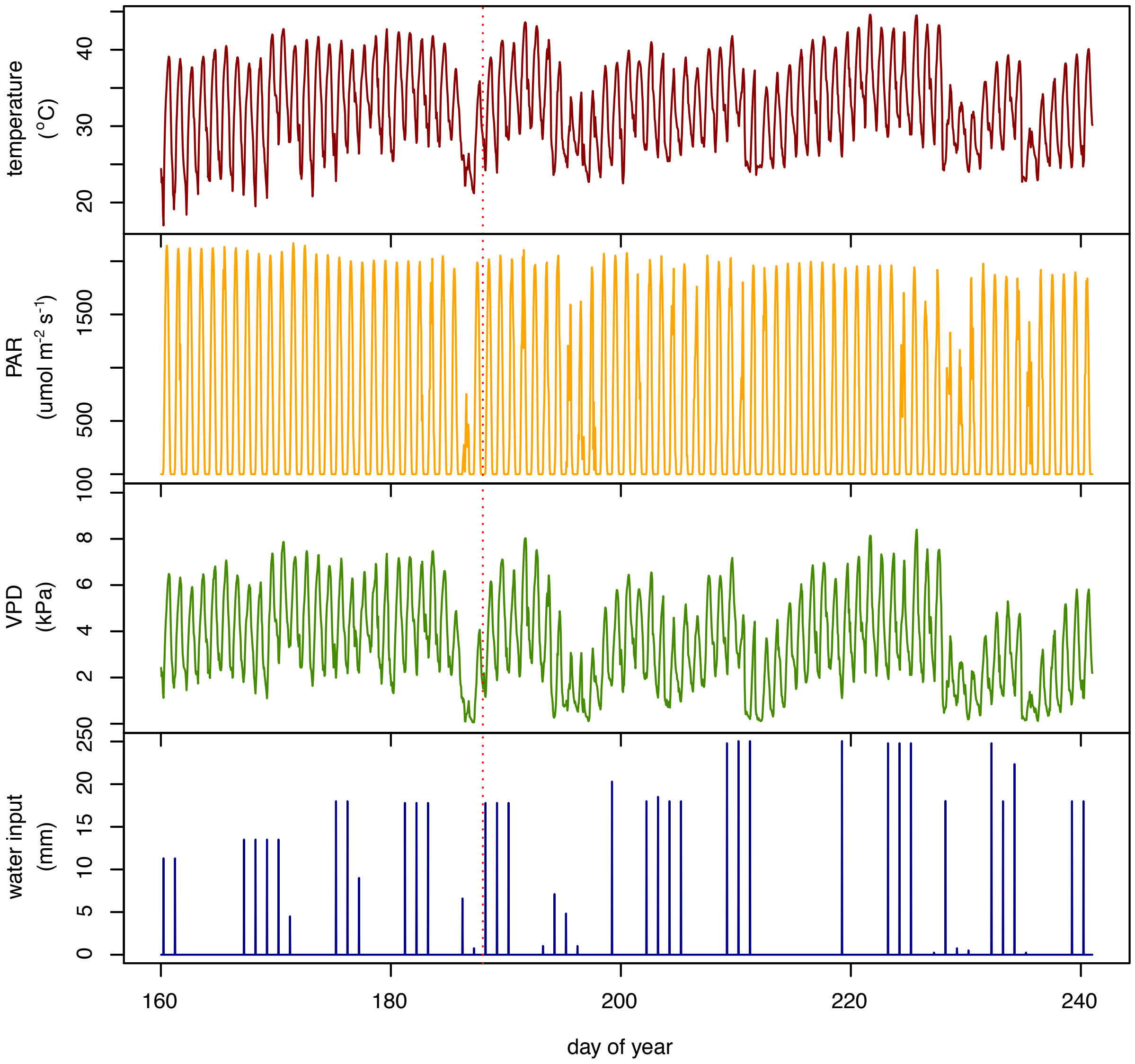

Fig. S6 Root parameterization. Distribution of roots across five soil layers were calibrated against available soil water content information by adjusting the proportion of roots via the parameter referred to as the "leaf area fraction." Briefly, the leaf area fraction per soil layer refers to the proportion of root area relative to the total root area. This total root area is determined based on an inputted ratio to the total leaf area. For more details, see hydraulic model of Sperry et al., 1998. Greater proportion of roots in a layer results in greater modeled water uptake from that layer. Displayed here are heat maps and visual representations of the final root proportions in the four simulations.

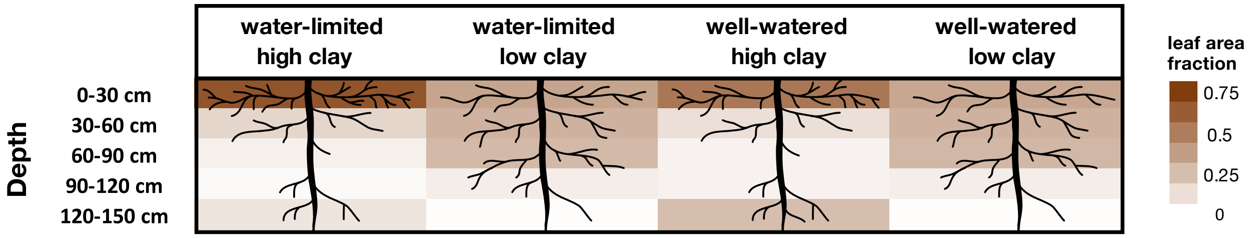

REFERENCE

Sperry JS, Adler FR, Campbell GS, Comstock JP. **1998**. Limitation of plant water use by rhizosphere and xylem conductance: results from a model. *Plant, Cell & Environment* **21**: 347–359.

Fig. S7 Modeled and observed canopy temperature. (a) Simulations are shown as time-courses with the grey shaded regions indicating the temperatures between the modeled sun canopy temperature and shade canopy temperatures. Points indicate observations collected from the High-Throughput Phenotyping platform and bars are standard errors of the mean from separate plots within each cell. One-to-one plots of **(b)** observed and modeled canopy temperatures and **(c)** observed and modeled temperature differences between canopy and air. Red lines denote the one-to-one relationship. All observations were from the evaluation carried out in 2012 and all canopy temperatures are presented in °Celsius.

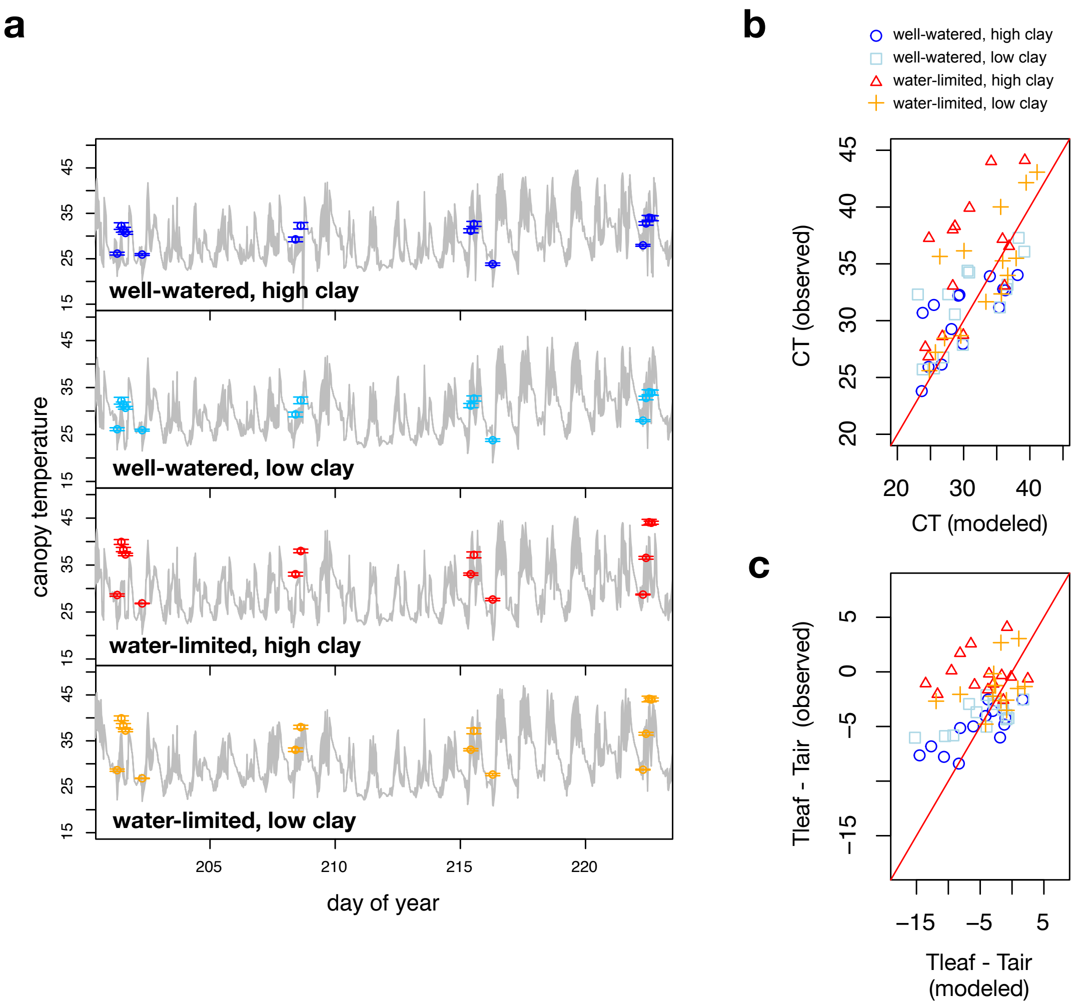

Fig. S8 Sensitivity of soil water potential to varying hydraulic vulnerability curves under three water input scenarios. Simulations with species (average) or genotype-specific vulnerability curves with average soil textures. X-axis shows the day of year in Julian Days. Start of differentiation in water inputs across the three scenarios (E1 [**a**], E2 [**b**] and E3 [**c**]) at day 188.

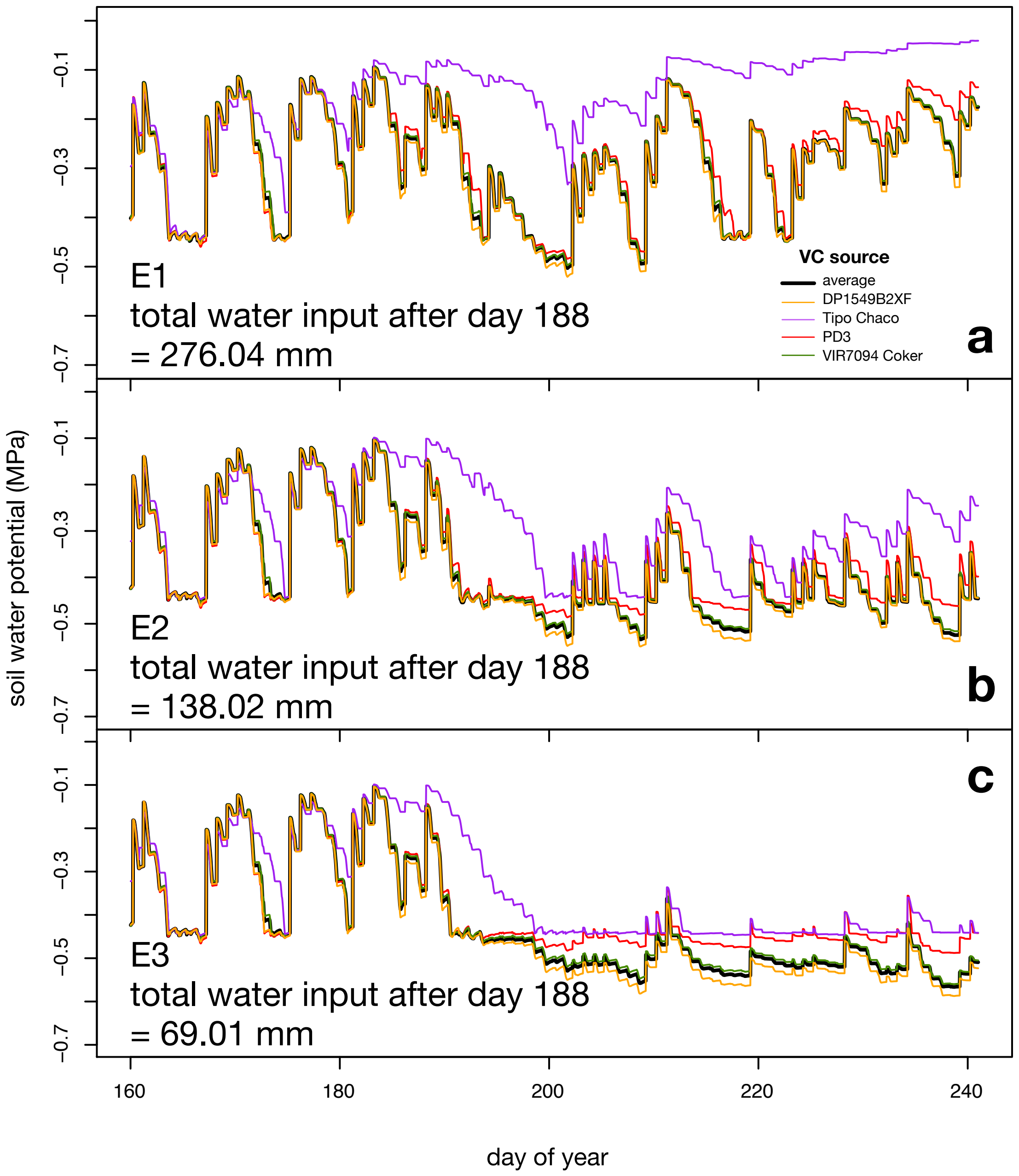

Fig. S9 Sensitivity of modeled leaf water potential to genotype-specific hydraulic vulnerability curves under three water input scenarios. (a, b, c) Full time-courses of leaf water potential and daily averages of **(d, e, f)** predawn (0300-0500 hours) and **(g, h, i)** approximate midday (1200-1400 hours) from simulations with species- or genotype-specific vulnerability curves with average soil textures. The three water input scenarios range from least water stressed (E1, top row) to most stressed (E3, bottom row). Total water inputs after day 188 when scenarios diverge are indicated in panels **a, b, c**. In boxplots: boxes indicate the interquartile range; upper whisker indicates min(maximum observation, third quartile plus 1.5 times the interquartile range); lower whisker indicates max(minimum observation, first quartile minus 1.5 times the interquartile range).

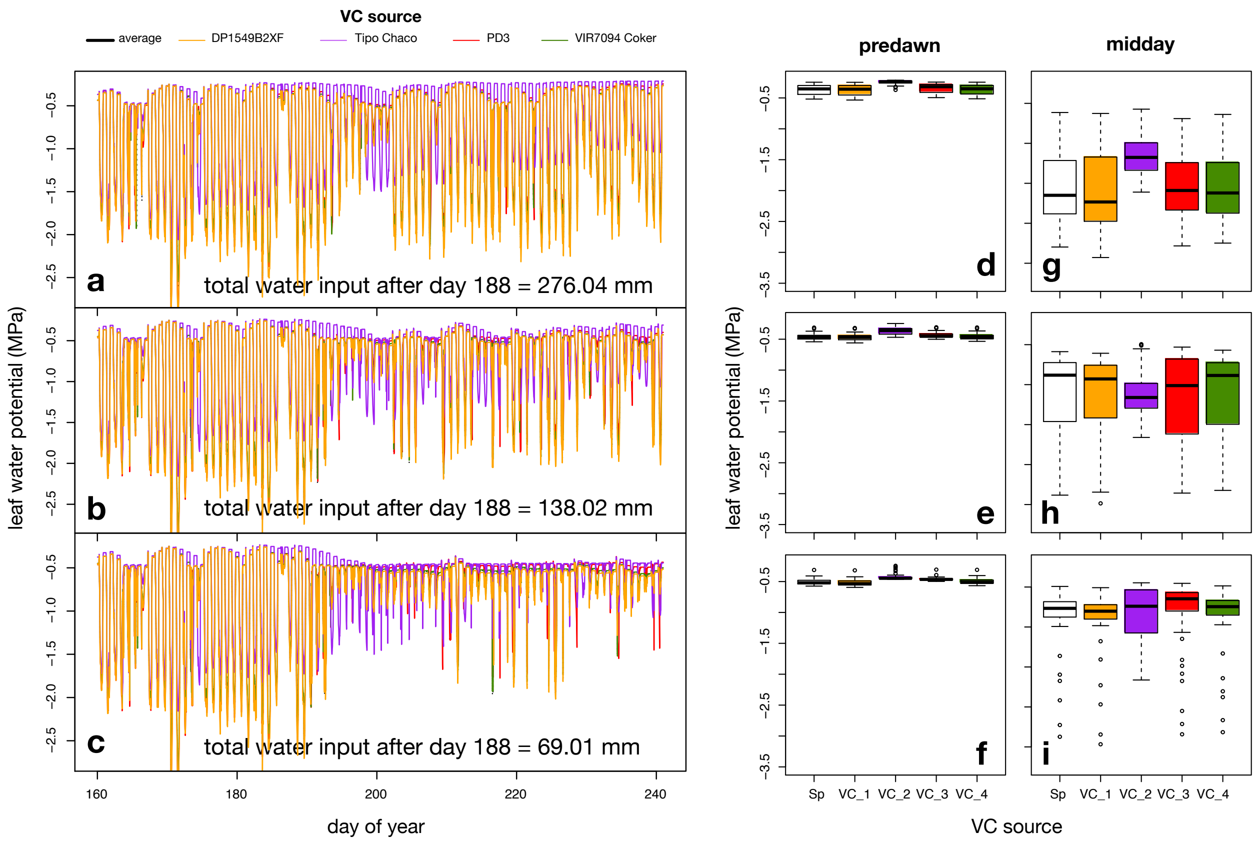

Fig. S10 Modeled leaf water potential response to contrasting genotype-specific hydraulic vulnerability curves and soil textures. Purple indicates simulations informed by Tipo Chaco's vulnerability curve; orange indicates simulations informed by DP1549's vulnerability curve. X-axis = day of year in Julian Days. Rows from top to bottom indicate least water stress (E1) to most water stress (E3). Column indicate high clay (left) and low clay (right) scenarios.

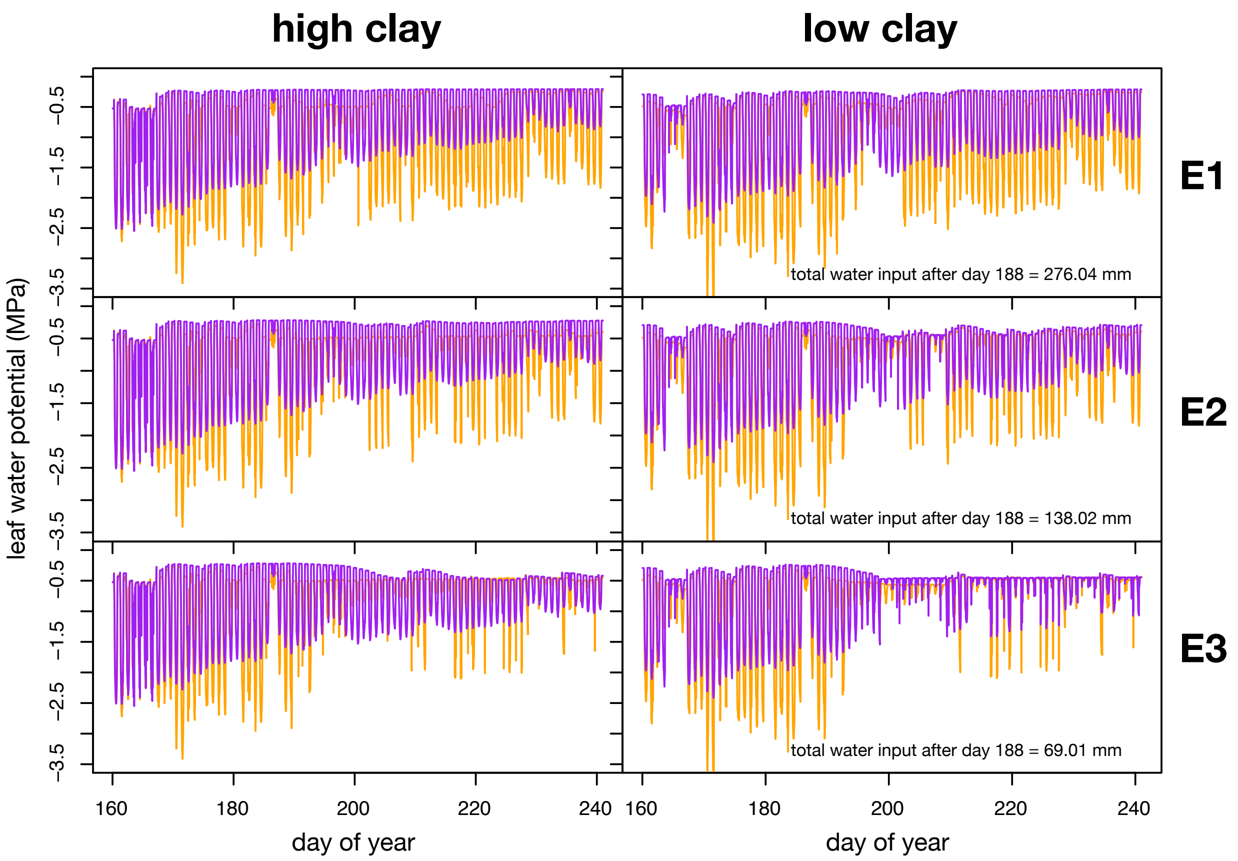

Tables S1, S2, S3 and S4 are found in separate Excel file

Methods S1 Experimental details at Maricopa Agricultural Center (MAC) in 2010-2012

Maricopa is in an irrigated production area that receives less than 100 mm of rainfall between April and September when cotton is grown and an ideal environment for drought studies. The TM-1 × NM24016 genetic mapping population was used for phenotyping stress-adaptive traits under high heat and water deficit conditions using a field-based, high-throughput phenotyping platform (Andrade-Sanchez *et al.*, 2014; Pauli *et al.*, 2016a). Briefly, the 95 RILs, parental lines, and commercial check varieties were evaluated in the field under two irrigation treatments, water-limited (WL) and well-watered (WW). Soils at the MAC field site are classified as Casa Grande sandy loam (fine-loamy, mixed, superactive, hyperthermic Typic Natrargids) (Post *et al.*, 1988). Each year the lines were arranged in an 11 × 10 α (0, 1) lattice design with two replications per irrigation treatment totaling 440 plots. Experimental plots were 8.8 m in length with a 0.61 m alley at the end and were thinned to a density of ~4.1 plants m^{-2} with an inter-row spacing of 1.02 m. Conventional cotton cultivation practices for the Southwest US were employed.

Planting occurred on May 7, 2010, April 27, 2011, and April 26, 2012. Once the crop was established using furrow irrigation, subsurface drip irrigation was used to water the crop starting in early June of each year and for the rest of the season. In late May in 2010, 2011, and 2012, a tractor-mounted soil sampler (Model 25-TS, Giddings Machine Company, Windsor, CO) was used to install neutron moisture probe access tubes to a depth of 1.6 m at 56 selected locations in the field. An equal number of access tubes were placed in the WW and WL treatment plots. Starting in early-June for each year, readings of volumetric soil water content (SWC) were taken on a nearly twice-weekly basis at 20cm increments starting at 10 cm depth (10, 30, 50, 70, 90, 110, 130, and 150 cm) using a field-calibrated neutron moisture probe (Model 503, Campbell Pacific Nuclear, CPN, Martinez, CA). Values of soil moisture at five depths in increments of 30 cm comparable to the five soil-root modules used in modeling were derived in the following manner: the 30 cm depth value was used directly for shallowest soil-root module, the average of the 50 cm and 70 cm values were used for the second soil-root module, the 90 cm depth was used for the third module, the average of the 110 cm and 130 cm was used for the fourth module, and the 150 cm depth value was used for the deepest (fifth) module. The scheduling of the WW irrigation treatment was carried out using a daily soil-water balance model computed using crop evapotranspiration (ETc) estimates determined from FAO-56 duel crop coefficient procedures (Allen *et al.*, 1998), metered irrigation depths, and precipitation data from the Arizona Meteorological (AZMET) Network weather station (Brown, 1989) located 270 m from the field site. Daily AZMET data for temperature, humidity, solar radiation, and wind speed were used to calculate daily Penman-Monteith grass-reference evapotranspiration (ETo). The cotton basal crop coefficient (Kcb) values were 0.15, 1.2, and 0.52 for the initial, mid-season, and end of season values, respectfully. These parameters were used to construct a daily FAO-56 Kcb curve using growth stage lengths developed locally by Hunsaker et al. (Hunsaker *et al.*, 2005) for a typical 155 day cotton season. Crop ETc was calculated by multiplying the daily crop coefficient by daily ETo. Additional crop and soil parameters used in calculating the daily soil water balance were taken from Hunsaker et al. (2005; table 3), except fraction of soil wetted by irrigations, which was reduced to 0.2 for the subsurface drip irrigation. The WL irrigation treatment was initiated when 50% of the plots were at first flower, after which this treatment received half of the irrigation amount relative to the WW treatment. Full details of the experimental design are presented by Pauli *et al.*, 2016a.

REFERENCES

Allen, RG, Pereira LS, Raes D, and Smith M. 1998. Crop evapotranspiration - guidelines for computing crop water requirements - FAO irrigation and drainage paper 56. *Food and Agriculture Organization of the United Nations, Rome* **300:** D05109.

Andrade-Sanchez P, Gore MA, Heun JT, Thorp KR, Carmo-Silva AE, French AN, Salvucci ME, White JW. **2014**. Development and evaluation of a field-based high-throughput phenotyping platform. *Functional Plant Biology* **41**: 68–79.

Brown, PW. 1989. Accessing the Arizona Meteorological Network (AZMET) by Computer in *Extension Report No. 8733*. University of Arizona, Tucson.

Hunsaker, DJ, Barnes EM, Clarke TR, Fitzgerald GJ, and Pinter JPJ. 2005. Cotton irrigation scheduling using remotely sensed and FAO-56 basal crop coefficients **48:** 1395-407

Pauli D, Andrade-Sanchez P, Carmo-Silva AE, Gazave E, French AN, Heun J, Hunsaker DJ, Lipka AE, Setter TL, Strand RJ. **2016**. Field-based high-throughput plant phenotyping reveals the temporal patterns of quantitative trait loci associated with stress-responsive traits in cotton. *G3: Genes, Genomes, Genetics* **4**: 865-879.

Post DF, Mack C, Camp PD, Suliman AS. **1988**. Mapping and characterization of the soils on the University of Arizona Maricopa Agricultural Center. Hydrology and Water Resources in Arizona and the Southwest. Tempe, Arizona USA: Arizona-Nevada Academy of Science.

Methods S2 Soil interpolation

To derive values for plots from which samples had not been directly collected, values for the proportions clay, sand, and silt were spatially interpolated using kriging. First, the proportions of each of the soil characteristics (clay, sand, and silt) were averaged across the five sampling depths to provide location means of clay, sand, and silt fractions for all 111 total soil sampling locations in the field. These means were then used to construct empirical variograms in the studied area as quantified by covariance as a function of distance, to assess the spatial relationship between sampling locations. Using the R package *gstat* (Pebesma, 2004), initial model parameters (i.e., nugget variance, sill, range, and variance structure) were subjectively generated based on the plot of the empirical variogram to establish a baseline fit. Next, the estimated model parameters were passed to the *gstat* auto-fitting function "fit.variogram" for further model optimization and parameter estimation with sample point weightings determined by N_j/h^2_j, where N is the number of point pairs and h is the distance between points. The final fitted models for each of the soil textures were visually inspected to ensure they were adequately capturing the spatial variance structure present in the field site. Finally, the optimized models for each soil texture were used to conduct block kriging whereby clay, sand, and silt percentages were predicted at the plot level for each georeferenced plot within the experimental site.

REFERENCE

Pebesma, EJ. 2004. Multivariable geostatistics in S: the gstat package. Computers & geosciences **30:** 683-691.

Methods S3 Determination of xylem vessel length distribution.

Xylem vessel length distribution was evaluated using the silicone injection method (Sperry et al. 2005; Christman et al. 2009). Stem samples from genotype DP1549B2XF were collected and shipped overnight to University of Utah in September 2019 as described in **Methods**. We selected six stem segments, each one coming from a different plant, for performing vessel length measurements. We trimmed the stem segments to 40 cm in length under water. The basal diameter of these segments (6.5 mm ± 0.5 SD) were equivalent in size to the 14 cm long segments used for vulnerability curve construction with the standard centrifuge method (Alder et al. 1997; Tobin et al. 2013). Stem segments were flushed with degassed filtered (0.2 μm) 10 Mm KCl solution for 1 h at 110 kPa to reverse native embolism. Then stem segments were infiltrated for 24 h at 30 kPa with a two component silicone (10:1 proportion of part A and B; RTV-141, Rhodia, Cranbury, NJ, USA) mixed with a fluorescent agent (1 drop per g of silicone of Ciba Uvitex OB, Ciba Specialty Chemicals, Tarrytown, NY, USA was dissolved at 1% w/w in chloroform; Christman et al. 2009). Samples were let to cure during a minimum of 48 h on a laboratory bench at room temperature (~23 ºC). Following, samples were rehydrated by immersion in water during 12 h for facilitating their sectioning. We used a sliding microtome (Reichert-Jung, Austria) for obtaining ca. 40 μm thickness cross-sections at 0, 9, 18, 35, 140, 280 and 380 mm from the silicone injection point. We placed the cross-sections on microscope slides. We captured 4 images per cross-section (one per cardinal point) under ultra-violet (UV) light with a fluorescence microscope at 4X magnification (Nikon Eclipse E600 connected to a SPOT digital camera). Silicone filled vessels are easy to identify since they glow blue under UV light (**Figure S4b,d**). We used Image-J (Rasband, W.S., ImageJ, U. S. National Institutes of Health, Bethesda, Maryland, USA, https://imagej.nih.gov/ij/, 1997-2018) to measure xylem area and count all silicone-filled vessels in the images. We calculated filled vessels density (number of filled vessels divided by xylem area, # mm^{-2}) for each distance from the injection point as the mean of the four images per cross-section per sample. We fitted the mean filled vessel density at each distance for each sample to an exponential decay Weibull function as described by Christman et al. 2009. We obtained the median and mean vessel distribution for each sample from the best fit Weibull curve as well as overall mean and median. We also fit all samples to a Weibull function to all samples at once for plotting the species exponential decay (**Figure S4e**) and fraction of vessels within 30 logarithmic vessel length classes (**Figure S4d**).

REFERENCES

Christman MA, Sperry JS, Adler FR. **2009**. Testing the 'rare pit'hypothesis for xylem cavitation resistance in three species of Acer. *New Phytologist* **182**: 664–674.

Sperry JS, Hacke UG, Wheeler JK. **2005**. Comparative analysis of end wall resistivity in xylem conduits. *Plant, Cell & Environment* **28**: 456–465.
